# Data on the expression and purification of Sestrin protein from *Dictyostelium discoideum*

**DOI:** 10.1016/j.dib.2019.103733

**Published:** 2019-03-08

**Authors:** S. Rafia, S. Saran

**Affiliations:** School of Life Sciences, Jawaharlal Nehru University, New Delhi, 110067, India

**Keywords:** Sestrin, *Dictyostelium*, Purification, Expression

## Abstract

The data present here is related to the research article entitled “Sestrin-like protein from *Dictyostelium discoideum* is involved in autophagy under starvation stress” [1]. The article provides data to show that *Dictyostelium* Sestrin share conserved amino acid residues, cysteine and aspartic acid with human Sestrin2. In human Sestrin2, these residues are involved in antioxidant activity along with AMPK activation and mTORC1 suppression [2]. The article provides the method of purification and expression of the fusion protein (Sesn + Igfp) driven by the endogenous *sesn* promoter and show prestalk expression during development.

**Table:** Specifications table

Subject area	Biology
More specific subject area	Developmental biology
Type of data	Figures, microscope, mass spectrophotometer, Table
Data format	Raw (microscopy), Analyzed (protein purification, Mass spectrophotometer)
How data was acquired	RT-PCR, Alpha Imager, fluorescence microscopy (SMZ1500, Nikon) Multiple sequence alignment using MUSCLE, Western blot, MALDI-TOF
Experimental factors	Development of wild type, *sestrin* mutants (overexpressors and knockout cells) for Eyfp analyses; cell lysate preparation for western blotting
Experimental features	Spatial expression pattern of *Ddsestrin* in wild type cells; Measurement of cell-typespecific (*ecmA, ecmB* and *pdsA*) gene expression in wild type and *sestrin* knockout cells; purification of Sestrin protein from Sestrin overexpressing cells
Data source location	School of Life Sciences, Jawaharlal Nehru University, New Delhi, India.
Data accessibility	All relevant data are within the article
Related research article	Rafia, S. Saran, S., 2019. Sestrin-like protein from *Dictyostelium discoideum* is involved in autophagy under starvation stress. Microbiol. Res. 220, 61–71.

**Table:** 

**Value of the data** • Sestrins are stress-inducible proteins that help maintain metabolic homeostasis and protect cells under stress conditions [3]. • Molecular mechanisms involved in the maintenance of metabolic stress, like nutrient-starvation are important research subjects and value to the scientific community. • In view of understanding the functions of Sestrin, here, we have identified, expressed and purified this protein from D. discoideum. • The data illustrates similar conserved residues between Dictyostelium Sestrin and human Sestrin2, suggesting the existence of possible similar functions, which could be analyzed in details by creating the mutations of these residues in Dictyostelium. • The data presented here shows that Sestrin in Dictyostelium is localized in the prestalk region that show developmental cell death in forming stalk cells under starvation (nutrient stress) conditions and involves autophagy [4], [5]. The data also describes the involvement of Sestrin in cell differentiation. The involvement of Dictyostelium Sestrin in autophagy would provide the basis for the identification of new pharmacological targets for drug discovery involving cell proliferation.

## 1 Data

Our aim was to identify, express and purify the Sestrin(s) from *Dictyostelium discoideum,* a protist whose development is initiated under starvation stress and thus require mechanism(s) to mobilize resources that could help maintain cellular homeostasis, which largely depends on autophagy. Accordingly, we found one Sestrin-like protein in *Dictyostelium* (DdSesn), which showed homology to human Sestrin2 (HsSesn2). Sestrins are involved in autophagy. Thus, studies in *Dictyostelium* would be beneficial in delineating the process of Sestrin-mediated autophagy as it naturally follows a caspase-independent cell death, thus preventing any crosstalks with apoptosis [6].

The conserved cysteine residue at position 221 in DdSesn is also present at position 125 in the catalytic region of HsSesn2. This residue present in HsSesn2 has similarity to bacterial AhpD family proteins that are involved in oxido-reductase activity and is essential for antioxidant functions. The conserved aspartate residues present at positions 525 and 526 in DdSesn is also present at positions 405 and 406 in HsSesn2. These residues present in HsSesn2 play a key role in the activation of AMPK and also the suppression of mTORC1 [2]. Based on the presence of these conserved residues it is speculated that DdSesn may exert a possible cytoprotective action via the antioxidant property and modulate the TOR signaling pathway (that is activation of AMPK and suppression of mTOR) [1].

The *Ddsestrin* gene was overexpressed, purified through nickel column and identified by MALDI-TOF. The fusion protein (Sestrin ORF under its own putative promoter using reporter gene, *igfp*) was localized in the prestalk region in all the multicellular structures developed.

## 2 Experimental design, materials and methods

### 2.1 Experimental design

To further evaluate the above idea regarding the functions of Sestrin, the single *sestrin-like* gene from *Dictyostelium* database was identified and the mutants (both overexpressors and knockout) were created.

### 2.2 Materials and methods

#### 2.2.1 Determination of conserved amino acids in DdSesn

**Fig. 1:**
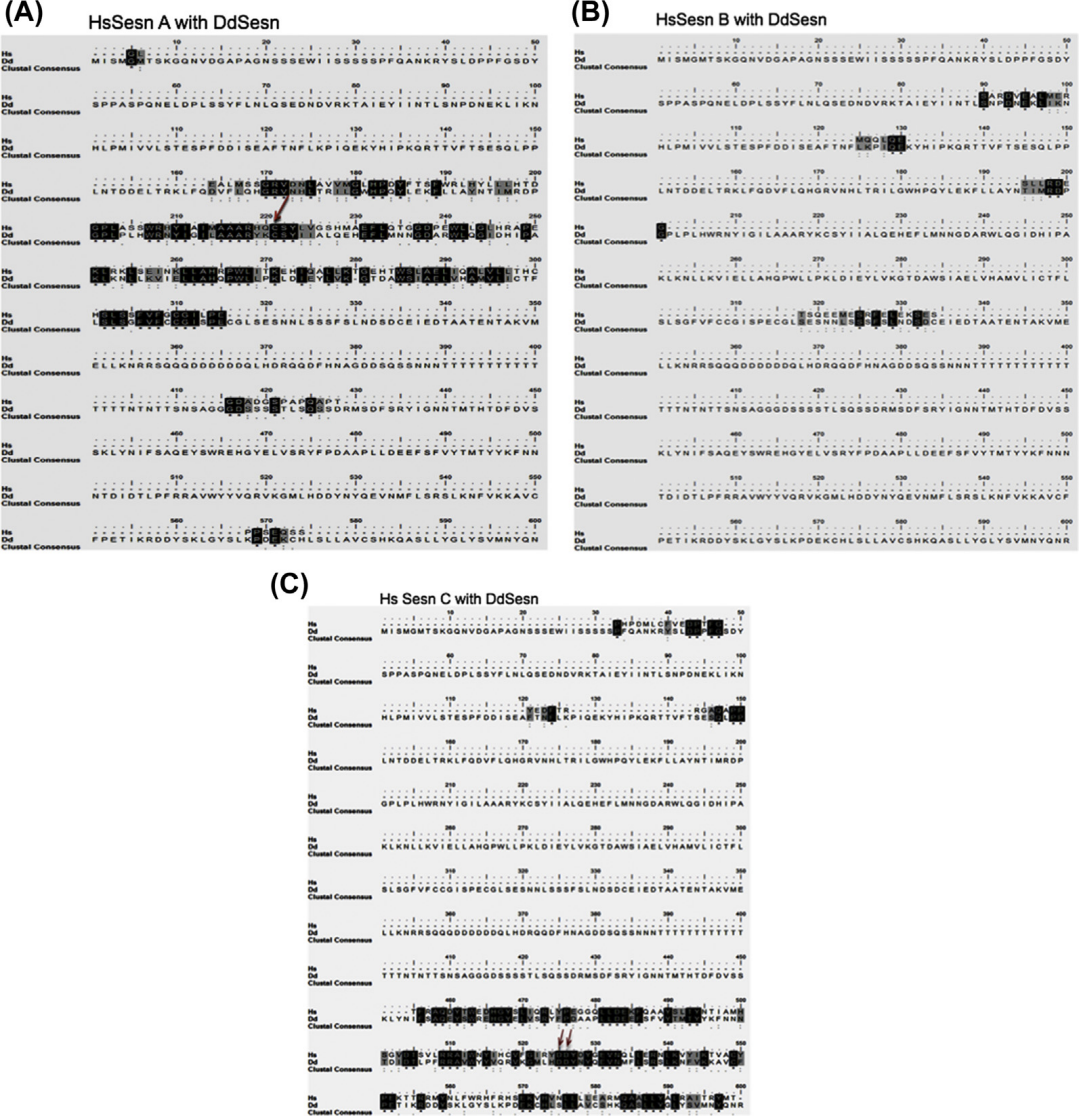
Multiple sequence alignment between human Sesn A, B and C domains with the full-length protein sequence of DdSesn. (A) Alignment with human Sesn A domain (position: 66–239). (B) Alignment with human Sesn B domain (position: 254–294) (C) Alignment with human Sesn C domain (position: 308–480). The conserved regions are indicated with black boxes. The orange arrows indicate conserved cysteine residues present in both DdSesn and HsSesn2 at 221 and 125 positions, respectively. Similarly, aspartate residues at 525, 526 and 405, 406 positions for DdSesn and HsSesn2, respectively are shown.

Based on the human Sestrin protein, DdSesn also has three domains (Sesn A-C). The MUSCLE program for the multiple sequence alignment between the full-length DdSesn and human Sesn2 domains (human: Sesn--A-domain: 66–239; B-domain: 254–294; C-domain: 308–480) was used (Fig. 1A–C).

#### 2.2.2 Purification of DdSesn protein

Protein was purified from [(*act15-sestrin-Eyfp*)/Ax2] cells, which expressed DdSesn fusion protein having enhanced green fluorescent protein (Eyfp) at the C-terminal and a 6x-His tag at the N-terminal. Nickel affinity gel column from Sigma Aldrich (cat No. P6611) was used for the purification of the protein. Protein was confirmed by immuno-blotting using anti-His antibody and further by MALDI-TOF.

**Fig. 2:**
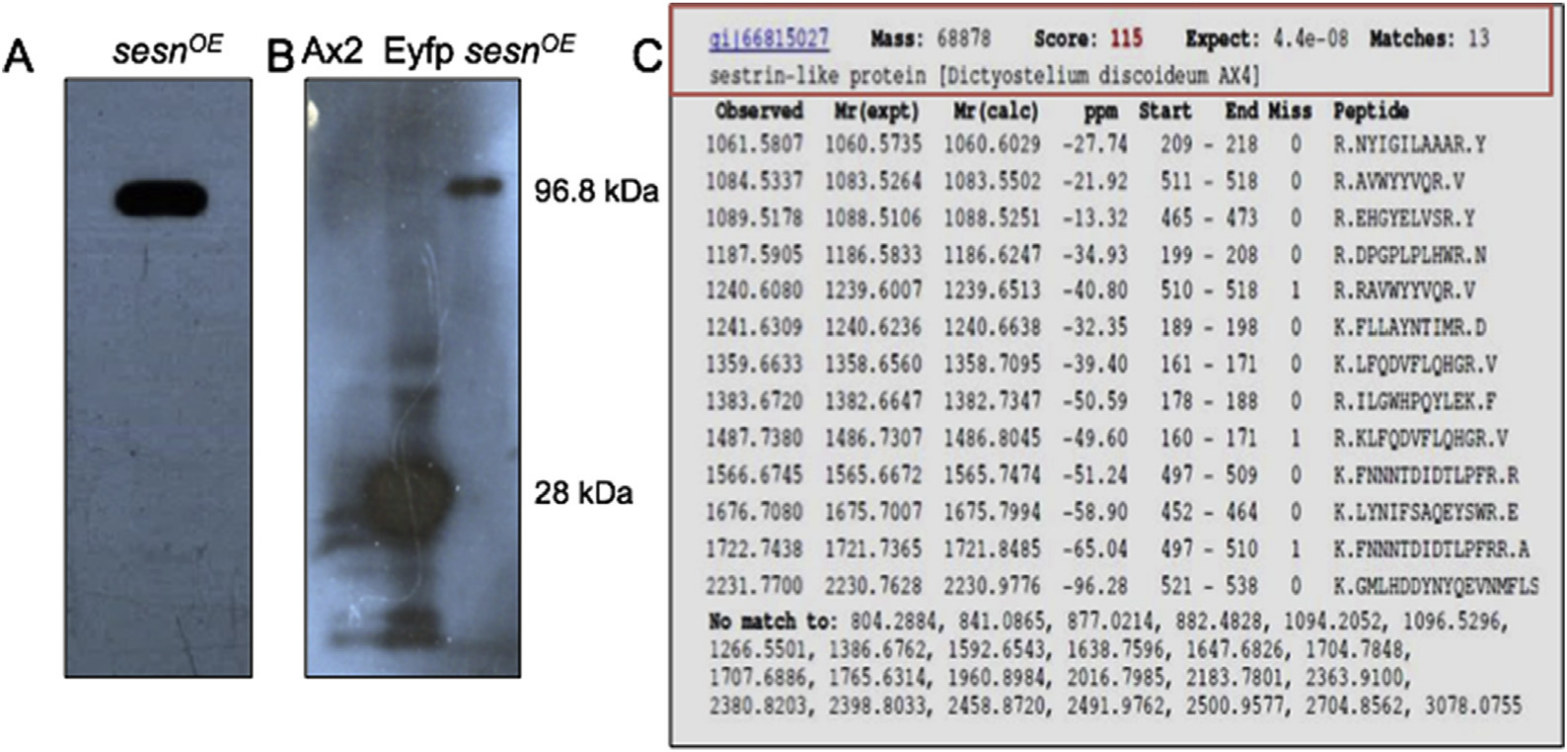
Purification and confirmation of DdSesn protein. (A) Sestrin protein was purified using the overexpressing [(*act15-sestrin-Eyfp*)/Ax2] strain and confirmed by western blotting using anti-His antibody. (B) Immunoblot using anti-His antibody (lane one: Ax2 (wild type), lane two: Eyfp vector only (positive control) and lane three: Sesn-Eyfp fusion protein. (C) Confirmation of the DdSesn protein by Matrix-assisted laser desorption/ionization time-of-flight (MALDI-TOF) mass spectrometry.

Briefly, cells (∼2 × 10^9^) from the log phase culture were harvested using chilled 1x KK2 (2.25 gm of KH_2_PO_4_ and 0.62 gm K_2_HPO_4_ dissolved in 1 liter water; pH 6.2) buffer and washed twice with the same buffer. Pellet obtained was re-suspended in 10 ml of cell lysis buffer having 1x protease inhibitor cocktail (Sigma-Aldrich, cat No. S8830) and 1% v/v Triton X-100 (50 mM Tris-HCl (pH 7.5), 150 mM NaCl, 5% Glycerol). The cell suspension was incubated for 30 min on ice with intermittent mixing and then centrifuged at 12,000 rpm at 4 °C for 20 min. The binding of clear supernatant with affinity gel was allowed by keeping the column at 4 °C under shaken conditions for approximately 3–4 hours. The affinity gel was pre-equilibrated with 2 ml of equilibration buffer (50 mM sodium phosphate, 300 mM sodium chloride) before binding to the supernatant. The mixture was allowed to settle in the purification column and flow throw was collected under gravity and stored for further use. Washing of beads (with three volumes of the beads) with wash buffer (50 mM sodium phosphate, 300 mM sodium chloride and 10 mM imidazole) was performed and the eluate was stored. Serial elution of bound protein using elution buffer (50 mM sodium phosphate, 300 mM sodium chloride with increasing (250 mM, 300 mM and 500 mM) concentrations of imidazole) was carried. The last elution was for overnight at 4 °C. Eluants having 6x-His tag were run on the SDS-PAGE followed by excision of the desired band and further sequenced using MALDI-TOF. Mascot search and further confirmation by western blotting using anti-His antibody (Sigma SAB1306084) was performed (Fig. 2).

#### 2.2.3 Spatial expression of DdSesn-Igfp in multicellular structures

**Fig. 3:**
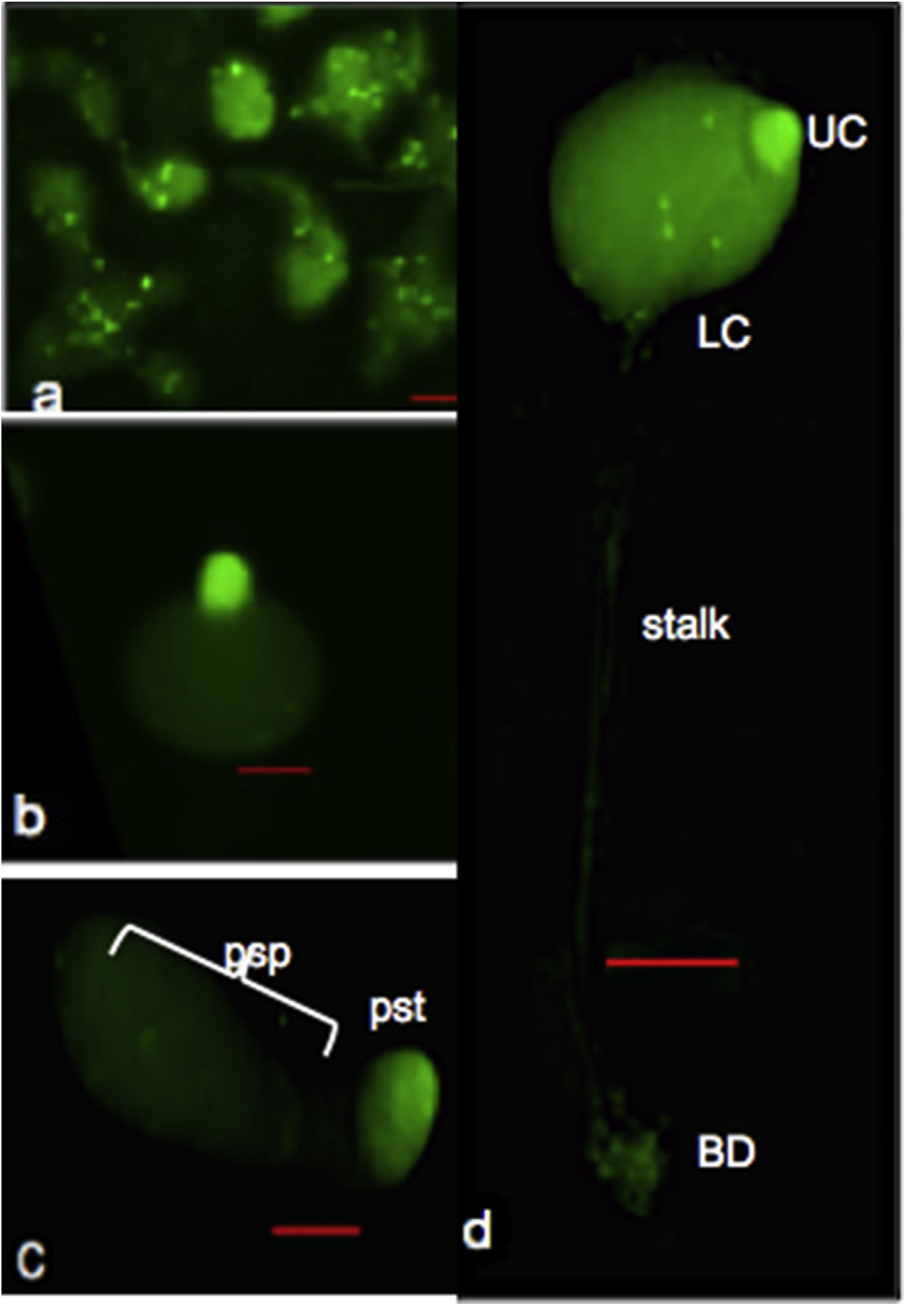
Spatial expression pattern of Sestrin fusion protein in wild type multicellular structures. Multicellular structures developed from [(*sesn/sesn-Igfp*]/Ax2 cells. Multicellular structures (a: aggregate; b: tipped mound; c: migrating slug; d: fruiting body) as visualized under fluorescence microscope. Expression largely seen in prestalk regions. [Scale bar: 100 μm; UC-upper cup; LC: lower cup; pst: prestalk; psp: prespore; BD: basal disc].

The spatial expression of Igfp driven by the *Ddsesn* promoter [p(*sesn/sesn-igfp*] in multicellular structures was analyzed. The full-length ORF of *Ddsesn* was fused at the C-terminal with the reporter *Igfp* (coding for a labile protein with half-life of 30 mins). Expression of fusion protein was driven under the endogenous *sestrin* promoter. For this, the region harboring the putative promoter (0.83 Kb, position: 1 to −828 bp) and the ORF (2.058 Kb, position: 1 to 2058 bp) was PCR amplified using the primers given in Table 1 and cloned in p(*CotB-Igfp*) vector (gift from Prof Pauline Schaap's lab) using XbaI and BglII restriction sites that replaces the original promoter. The construct [p(*sesn/sesn-/Igfp*] was transformed into Ax2 cells by electroporation [7] and transformants were selected on G418 (maximum: 40 μg/ml, G418). The positive clones were grown and allowed to develop on non-nutrient agar plates and photographs of various developmental structures were taken on SMZ1500 Nikon microscope. This is shown in Fig. 3.

**Table 1:** List of oligonucleotides used in this study.

Gene name	Primers 5′→3′	Genomic position
*Sestrin (Pr + ORF)*	FP ATGCTCTAGAAACGGTAGTTGCGCCTCTCAA	−828 to −808
	RP AGGAAGATCTCTAGCTTGTTTATGAGAACAA	2038 to 2058
*ecmA*	FP AACTAAGCTTCAAATCAACAGGTGTCACTCATACCC	1684-1709
	RP TTACCTCGAGACAACCAGTTAAGTCATCGCAAGAATC	2609-2635
*ecmB*	FP GGTCGGGATCCAATAACGCATGTACTGAAGATAAATG	1494-1519
	RP TATTCTCGAGTTAATTGGAGTGTGGACACAA	2683-2707
*pdsA*	FP GATAAGGATCCCCAGTTTGTGCTTCAGTAGATGTC	94-117
	RP ACTTCTCGAGGTTGTTGTTGATGTTTGGGATGG	415-434
*m/A*	FP TGAATTGAAGTCTGAGTAAACGG	1795-1817
	RP TAGATAGGGACCAAACTGTCTCAC	3065-3042

#### 2.2.4 RT-PCR of cell-type specific genes

**Fig. 4:**
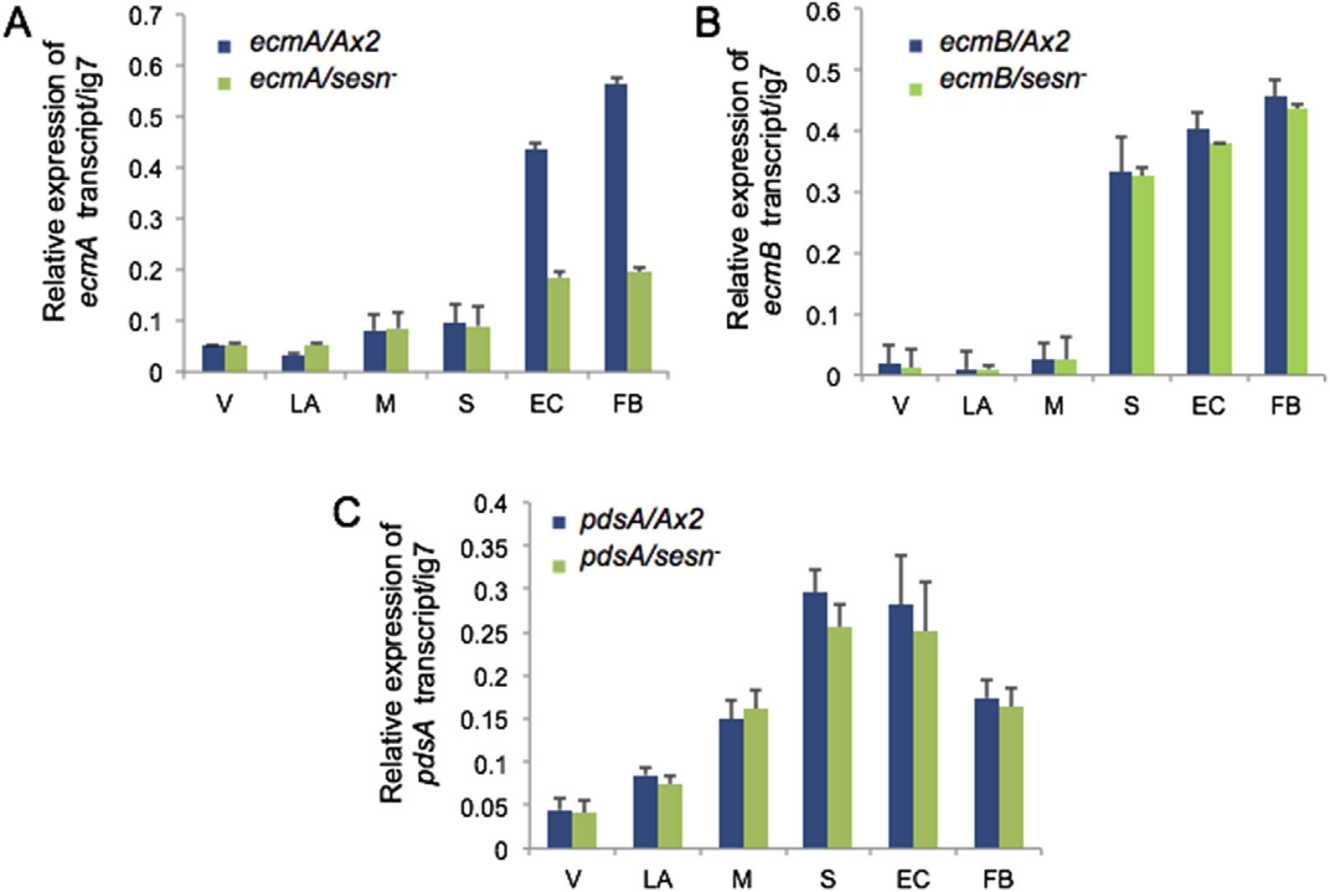
mRNA expression patterns of cell-type specific genes as studied by semi-quantitative RT-PCR. (A) Relative abundance of *ecmA* (952 bp), (B) *ecmB* (1214 bp) and (C) *pdsA* (344 bp) transcripts in Ax2 and *sesn*^*-*^ cells during development. *ig7* (*rnlA*, 720 bp) was taken as an internal control. (V-vegetative; LA-loose aggregate; M-mound; EC-early culminant; FB-fruiting body).

The mRNA levels were monitored by semi-quantitative RT-PCR using primers as shown in Table 1 [1]. We have analyzed both prestalk (*ecmA* and *ecmB*) and prespore (*pdsA*) specific genes in the wild type and *sestrin* knockout cells collected from different developmental stages [8]. The data is shown in Fig. 4.

## Funding sources

SS thanks partial grants from DST-PURSE, UPE-II and FIST-II. SR thanks UGC for research fellowship.
